# Prevalence of metabolic syndrome and its components in Brazilian adolescents: a systematic review and meta-analysis

**DOI:** 10.1590/1984-0462/2023/41/2021145

**Published:** 2022-07-06

**Authors:** Miguel Henrique Pereira de Paiva, Valberto Alencar Miranda, Ana Raquel Soares de Oliveira, Kyria Jayanne Clímaco Cruz, Regina Maria Sousa de Araújo, Karla Andrade de Oliveira

**Affiliations:** a Universidade Federal do Piauí, Teresina, PI, Brazil.

**Keywords:** Metabolic syndrome, Prevalence, Adolescent, Brazil, Síndrome metabólica, Prevalência, Adolescente, Brasil

## Abstract

**Objective:**

To determine the prevalence of metabolic syndrome (MS) and its components among Brazilian adolescents.

**Data source:**

Databases, such as LILACS, MEDLINE, and SciELO, were searched for original cross-sectional studies published between 2010 and 2021. The inclusion criteria were determined based on the mnemonic CoCoPop — Condition, Context, and Population: studies determining the prevalence of MS and its components (condition) in the general population of Brazilian adolescents, female and male (population), enrolled in public or private schools in rural or urban areas (context). Reviews, editorials, and articles that did not directly relate to the prevalence of MS or that included non-adolescent age groups or groups with specific health conditions (obesity/overweight and others) were excluded.

**Data synthesis:**

A total of 15 studies, including 43,227 adolescents, were identified. MS prevalence (95% confidence interval [95%CI]) was 2.9% (2.65–3.18) and 2.4% (1.90–2.90) (p<0.001) in males and females, respectively, by using the International Diabetes Federation (IDF) criteria. There was a significant difference in MS prevalence among Brazilian regions (Q=24.7; p<0.001). The lowest MS prevalence (95%CI) was determined for North Region of Brazil, 1.8% (1.52–2.13), and the highest for Northeast Region of Brazil, 2.9% (2.62–3.23). Regarding MS components, a higher prevalence (95%CI) was found for low high-density lipoprotein (HDL), 22.1% (12.49–36.17), followed by abdominal obesity, 11.0% (8.05–14.94), and arterial hypertension, 10.3% (7.84–13.48).

**Conclusions:**

This study allowed the determination of the prevalence of MS and the MS components in Brazilian adolescents, highlighting relevant aspects to be addressed on public health management.

## RESUMO

**Objetivo:**

Determinar a prevalência da síndrome metabólica (SM) e seus componentes em adolescentes brasileiros.

**Fontes de dados:**

Realizaram-se buscas nas bases de dados Literatura Latino-Americana e do Caribe em Ciências da Saúde (Lilacs), Medical Literature Analysis and Retrieval System Online (Medline) e Scientific Electronic Library Online (SciELO) por estudos transversais originais publicados entre 2010 e 2021. Os critérios de inclusão foram determinados pelo Mnemônico CoCoPop — Condição, Contexto e População: estudos que determinam a prevalência de SM e seus componentes (condição) na população geral de adolescentes brasileiros, feminino e masculino (população), matriculados em escolas públicas ou privadas em áreas rurais ou urbanas (contexto). Revisões, editoriais, artigos não relacionados com a prevalência de SM ou que incluíssem grupos etários não adolescentes ou com condições de saúde específicas (obesidade/sobrepeso e outros) foram excluídos.

**Síntese dos dados:**

Quinze estudos, com 43.227 adolescentes, foram identificados. A prevalência de SM (intervalo de confiança — IC95%) no sexo masculino foi de 2,9% (2,65–3,18) e no feminino foi de 2,4% (1,90–2,90) (*p*<0,001), pelos critérios da Federação Internacional de Diabetes (IDF). Houve diferença significativa na prevalência de SM entre regiões brasileiras (Q=24,7; *p*<0,001). A menor prevalência de SM (IC95%) foi encontrada em região Norte, com 1,8% (1,52–2,13), e a maior na região Nordeste, com 2,9% (2,62–3,23). Em relação aos componentes da SM, maior prevalência (IC95%) foi encontrada para lipoproteína de baixa densidade (HDL) baixa (22,1%, 12,49–36,17), seguida de obesidade abdominal (11,0%, 8,05–14,94) e hipertensão arterial (10,3%, 7,84–13,48).

**Conclusões:**

Este estudo permitiu determinar a prevalência da SM e seus componentes em adolescentes brasileiros, destacando aspectos relevantes a serem abordados na gestão da saúde pública.

## INTRODUCTION

Metabolic syndrome (MS) is characterized by a cluster of risk factors for the development of cardiovascular diseases, including abdominal obesity, high blood pressure, low serum high-density lipoprotein cholesterol (HDL-C), insulin resistance, and high serum triglyceride (TG).^1,2^

Difficulties that include physical and metabolic changes of adolescence and the influence of puberty variations in the parameters used, such as body mass index (BMI) and abdominal circumference, make it difficult to know the prevalence of this pathology in this population.^3–5^ In addition, there are no consensus criteria for a standardized definition of MS in children and adolescents. The International Diabetes Federation (IDF) defined the criteria for MS in children and adolescents according to age,^6^ and the use of this definition was suggested by the Brazilian Diabetes Society.^7^

The importance of identification of MS and/or its components in this age group is justified by the increasing prevalence of obesity and its association with other components of MS, such as diabetes mellitus, arterial hypertension, and dyslipidemias, which can persist into adulthood, increasing the risk of cardiovascular diseases and other chronic diseases.^8–10^

We conducted a preliminary search for systematic reviews on MS prevalence in Brazilian adolescents using the PubMed database, and it returned a 2010 review by Tavares et al.^11^ In this work, a few population-based studies, mainly from the Southeast Region of Brazil, were available, as well as a high heterogeneity due to the use of several criteria for MS definition in the selected studies was identified, limiting the determination of MS prevalence in adolescents in Brazil. In addition, the absence of a systematic review and meta-analysis on the prevalence of MS and its components in Brazilian adolescents evokes the need for such a study. Thus, this study aimed to systematically quantify the prevalence of MS and its components in adolescents in Brazil from 2010 to 2021.

## METHOD

We conducted a systematic and meta-analytical review of the literature following the instructions provided by Moher et al.^12^ and the guidelines of the Joanna Briggs Institute (JBI),^13^ two validated tools for study selection and analysis, respectively. This last one is recommended for systematic reviews of prevalence. The hypothesis was: MS is prevalent among adolescents in all Brazilian regions.

The inclusion criteria were determined based on the mnemonic CoCoPop — Condition, Context, and Population, following the JBI recommendation for reviews assessing prevalence/incidence data.^13^ The inclusion criteria were as follows: original cross-sectional studies published in national or international journals (in Portuguese, English, or Spanish) between 2010 and July 2021; and studies determining the prevalence of MS and its components (condition) in the general population of Brazilian adolescents, females and males (population), enrolled in public or private schools in rural or urban areas (context). Reviews, editorials, as well as articles that did not directly relate to the prevalence of MS or that included non-adolescent age groups or groups with specific health conditions (obesity/overweight and others) were excluded.

The search for studies occurred from May 2020 to July 2021, being carried out by two researchers independently. The databases searched were LILACS, MEDLINE, and SciELO. A third reviewer participated in the decision of articles’ inclusion/exclusion when necessary.

The following search strategies were employed:

- LILACS: (tw:(metabolic syndrome)) AND (tw:(adolescent*)) AND (tw:(prevalence)) OR (tw:(frequency)) AND (tw:(brazil*))
- MEDLINE: (((((((metabolic syndrome) AND (adolescent)) AND (prevalence)) AND (brazil)) OR (metabolic syndrome)) AND (adolescent)) AND (frequency)) AND (brazil)
- SciELO: (metabolic syndrome) AND (adolescent) AND (prevalence) AND (brazil) OR (metabolic syndrome) AND (adolescent) AND (frequency) AND (brazil).

The quality analysis of the studies was carried out according to the JBI Critical Appraisal Tool,^13^ consisting of 10 questions: representative sample, appropriate recruitment, adequate sample size, appropriate description of the subjects, adequate data coverage of the identified sample, reliability and objectivity on condition measurements, appropriate statistical analysis, and identification and consideration of confounding factors/subgroups/differences. Each question was considered independently during the analysis of the risk of bias and was answered with “Yes,” “No,” or “Unclear.”

The quality analysis of the studies was conducted by two independent reviewers. The discussion with a third reviewer was requested when a disagreement occurred.

A data collection instrument containing the following topics was used: (1) details of the study: year of publication, first author, and journal; (2) study methods: location (state), context (urban or rural area, public or private school), study design, characteristics of the study population, comorbidities, criteria used for MS diagnosis, blood pressure measurement method, biochemical analysis method, and abdominal obesity measurement method; and (3) results: prevalence of MS and its components.

Data were synthesized by conducting a meta-analysis. Quantitative analyses of the MS prevalence and its components were performed considering the IDF criterion.

A subgroup analysis considering different criteria for the diagnosis of MS was performed for the comparison of MS prevalence.

The meta-analysis was performed using a random-effect model (random intercept logistic regression model) with transformed proportions (logit transformation). Maximum-likelihood estimation was used as an estimator for τ^2^. Heterogeneity was assessed using I^2^ statistic,^14^ τ^2^, and Cochran’s Q tests. Subgroup analysis, defined *a priori*, considering different Brazilian regions, was used to access the source of heterogeneity and influence analysis of individual studies on the overall effect.

Sensitivity analyses were performed to assess the risk of bias, in which subgroup analysis for studies carried out in public schools versus private schools; subgroup analysis for studies that used a sphygmomanometer versus studies that used automatic equipment to measure blood pressure; studies grouped according to the abdominal obesity measurement method; studies grouped according to sample coverage (group of studies that reported considerable losses of eligible participants or that did not report losses vs. group formed by the other studies); sensitivity analyses by excluding studies that did not use a random selection of schools; and sensitivity analysis by exclusion of articles considered at high risk of bias after quality analysis and sensitivity analysis by exclusion of studies carried out in rural areas.

Publication bias was accessed by funnel plot, using the rank correlation test^15^ to test funnel plot asymmetry.

Using the IDF criteria, analyses by sex were performed based on data from nine studies. Proportions were analyzed using the chi-square test.

Analyses were performed using the R software (Rstudio® version 4.0.1).^16^

The objectives, inclusion/exclusion criteria, and methods of this review were documented in PROSPERO protocol (CRD42021222934).

## RESULTS

**Figure 1:**
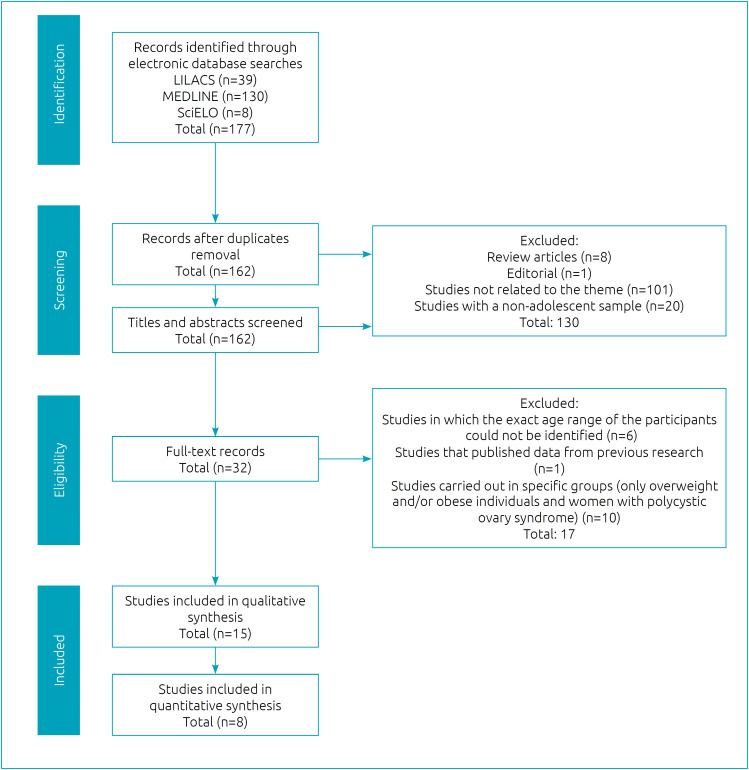
Flowchart of records retrieved, screened, and included in the systematic review.

Source: adapted from Moher et al.^12^

A total of 15 studies proceeded to the quality analysis, of which 8 studies that defined MS by using the IDF criteria were included in quantitative analysis. The process of identification, screening eligibility, and inclusion of studies is detailed in Figure 1.

One study for 2010;^17^ two studies for each of 2011,^18,19^ 2013,^20,21^ 2016,^22,23^ and 2017;^24,25^ and three studies for 2018^26–28^ and 2019^29–31^ were retrieved.^23^ Data from this study were extracted by Brazilian regions to determine the combined prevalence of MS in this meta-analysis. The other studies were unicentric, carried out in nine states, namely, Bahia (n=1), Espírito Santo (n=1), Maranhão (n=1), Mato Grosso do Sul (n=1), Minas Gerais (n=3), Paraná (n=2), Piauí (n=3), Rio de Janeiro (n=1), and Rio Grande do Sul (n=1). The studies had a minimum sample population of 85 individuals^25^ and a maximum of 37,504 individuals,^23^ with participants aged 10–19 years. The proportion of female and male adolescents was 59.78 and 40.22%, respectively. The minimum prevalence of overweight/obesity was 14.35%, ^22^ and the maximum prevalence was 52.8%.^19^ Table 1 presents the general characteristics of the studies included in the qualitative analysis.

**Table 1:** Studies included in the qualitative analysis and the prevalence of metabolic syndrome for each study retrieved followed by their respective criteria.

Study	Age group (year)	School/Zone	Sample (n)	Prevalence of MS (%)
Quintão et al.^17^	16–19	Public and private/urban area of Minas Gerais (MG)	172	1.2 IDF (2005)^6^
Alvarez et al.^18^	12–19	Public/urban area of Rio de Janeiro (RJ)	577	6.0 Ford et al.;^37^ 1.1 Viner et al.;^32^ 1.6 IDF (2005)^6^
Stabelini Neto et al.^19^	12–18	Urban area of Paraná (PR)	582	6.7 Cook et al.^35^
de Sousa et al.^20^	11–18	Public and private/urban area of Bahia (BA)	250	21.6 Ferranti et al.^36^
Furtado Neto e Ribeiro^21^	12–17	Public and private/urban area of Maranhão (MA)	468	12.2 Cook et al.^35^
Granjeiro et al.^22^	10–17	Public/urban area of Minas Gerais (MG)	202	0.50 Viner et al.^32^
Kuschnir et al.^23^	12–17	Public and private/urban area of Brazil: 26 States and Distrito Federal (DF)	37,504	2.6 IDF (2005):^6^ North: 1.8; Northeast: 2.9; Midwest: 2.8; Southeast: 2.6; South: 4.1
Assis et al.^24^	15–17	Public and private/urban area of Minas Gerais (MG)	302	4.0 IDF (2005)^6^
Pani et al.^25^	11–15	Public/urban area of Espírito Santo (ES)	85	2.4 Faria et al.^33^
dos Santos et al.^26^	12–18	Public/urban area of Mato Grosso do Sul (MS)	274	4.7 IDF (2005)^6^
Nobre et al.^27^	10–19	Public/urban area of Piauí (PI)	716	3.2 Cook et al.^35^
Reuter et al.^28^	10–17	Urban and rural areas of Rio Grande do Sul (RS)	1,200	1.9 Cook et al.;^35^ 5.0 Ferranti et al.;^36^ 2.1 IDF (2005)^6^
Guilherme et al.^29^	10–14	Public and private/urban area of Paraná (PR)	241	1.7 IDF (2005);^6^ 3.3 Cook et al.;^35^ 17.4 Ferranti et al.^36^
Lustosa et al.^30^	14–19	Public and private/urban area of Piauí (PI)	327	3.4 IDF (2005)^34^
Mendes et al.^31^	14–19	Public and private/urban area of Piauí (PI)	327	7.0 NCEP-ATP III

IDF: International Diabetes Federation; NCEP-ATP III: National Cholesterol Education Program Adult Treatment Panel III.

This review identified six criteria used for MS diagnosis in adolescents in the analyzed studies: IDF (n=8); studies that used the World Health Organization (WHO) criteria adapted by Viner et al.^32^ (n=2) and Faria et al.^33^ (n=1); National Cholesterol Education Program Adult Treatment Panel III (NCEP-ATP III)^34^ (n=1); and NCEP-ATP III adapted by Cook et al.^35^ (n=5), Ferranti et al.^36^ (n=3), and Ford et al.^37^ (n=1).

All studies analyzed were cross-sectional studies. Regarding the recruitment method, most studies (n=5) carried out cluster sampling, and for the remaining three studies, the sampling process was not clear, and one of these studies selected a specific region of the city. Few studies (n=3) reported a detailed description of the participants. All studies were performed with a representative sample size, but some (n=3) did not specify the effect of the design on the sample calculation and reported considerable losses of eligible participants (>24.0%); and one study did not report any loss.

Concerning the measurement methods, only four studies clarified that they were carried out by trained people. Specifically, blood pressure was measured using a sphygmomanometer (n=2) and automatic monitors (n=4), and two studies did not report the measurement method of blood pressure. Regarding the measurement of abdominal obesity, studies reported the measurement of waist circumference at the midpoint between the last rib and the top of the iliac crest (n=3), the smallest value of waist circumference between the last rib and the top of the iliac crest (n=3), and the circumference measurement immediately above the iliac crest (n=1); and for one study, it was not possible to determine the measurement methodology.

Regarding the measurement of biochemical parameters, a study did not make clear of the methodology used; most studies (n=7) reported the analyses carried out in laboratories, of which four studies specified enzymatic colorimetric assays. Three studies were assigned “No” or “Unclear” answers for three questions in this tool, standing out among the others for presenting a higher risk of bias. Table 2 summarizes the results of the quality analysis of the studies.

**Table 2:** Quality analysis of included studies in the qualitative and/or quantitative analyses (n=15).

Study	Was the sample representative of the target population?	Were participants recruited in an appropriate way?	Was the sample size adequate?	Were the study subjects and the setting described in detail?	Was the analysis conducted with sufficient coverage of the sample?	Were objective, standard criteria used for the measurement?	Was the condition measured reliably?	Was there appropriate statistical analysis?	Are all important confounding factors/subgroups identified and accounted for?	Were subpopulations identified using objective criteria?
Quintão et al.^17^	Yes	Unclear	Yes	No	Unclear	Yes	Yes	Yes	Yes	Yes
Alvarez et al.^18^	Yes	Yes	Yes	No	Unclear	Yes	Unclear	Yes	Yes	Yes
Stabelini Neto et al.^19^	Yes	No	Yes	No	Yes	Yes	Yes	Yes	Yes	Yes
de Sousa et al.^20^	Yes	Yes	Yes	Yes	Unclear	Yes	Unclear	Yes	Yes	Yes
Furtado Neto e Ribeiro^21^	Yes	Yes	Yes	Yes	Unclear	Yes	Unclear	Yes	Yes	Yes
Granjeiro et al.^22^	Yes	Yes	Yes	Yes	Yes	Yes	Unclear	Yes	Yes	Yes
Kuschnir et al.^23^	Yes	Yes	Yes	No	Yes	Yes	Yes	Yes	Yes	Yes
Assis et al.^24^	Yes	Unclear	Yes	No	Yes	Yes	Yes	Yes	Yes	Yes
Pani et al.^25^	Yes	Unclear	Yes	No	Unclear	Yes	Yes	Yes	Yes	Yes
dos Santos et al.^26^	Yes	Unclear	Yes	Yes	Unclear	Yes	Unclear	Yes	Yes	Yes
Nobre et al.^27^	Yes	Unclear	Yes	Yes	Unclear	Yes	Unclear	Yes	Yes	Yes
Reuter et al.^28^	Yes	Yes	Yes	Yes	Yes	Yes	Unclear	Yes	Yes	Yes
Guilherme et al.^29^	Yes	Yes	Yes	No	Unclear	Yes	Yes	Yes	Yes	Yes
Lustosa et al.^30^	Yes	Yes	Yes	Yes	Yes	Yes	Unclear	Yes	Yes	Yes
Mendes et al.^31^	Yes	Unclear	Yes	Yes	Yes	Yes	Unclear	Unclear	Yes	Yes

Sensitivity analyses, to assess the risk of bias, were performed by excluding articles in which the sampling was non-random or not clear (n=3); exclusion of a study carried out in rural areas (n=1); and exclusion of studies considered to be at high risk of bias (n=3), with no significant effect on MS prevalence by the chi-square test.

Subgroup analyses were performed for groups of studies in public and private versus public schools (there were no studies performed exclusively in private schools), with no significant differences between groups (p=0.974), for groups of different methods of measurement of blood pressure (p=0.943), for groups of different methods of measurement of abdominal obesity (minimum circumference, average circumference, and unclear) (p=0.172), and between groups of studies that reported considerable losses from eligible participants or did not report losses, and the other studies (p=0.368).

An initial quantitative analysis by subgroup, according to criteria used to define MS, including 15 studies, resulted in significantly different (p<0.001) MS prevalence (95% confidence interval [CI]) and I^2^ (%) for IDF, Ferranti et al., and Cook et al.as follows: 2.6% (2.24–2.92; I^2^ 67.1%), 12.5% (5.90–24.67; I^2^ 95.7%), and 4.5% (2.44–8.05; I^2^ 92.3%), respectively.

**Figure 2:**
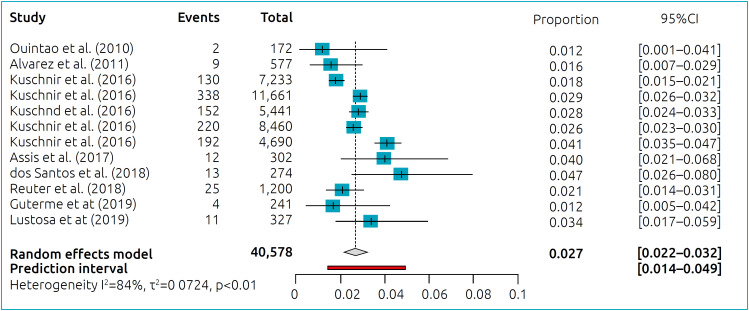
Meta-analysis of cross-sectional studies: prevalence of MS including 40,578 Brazilian adolescents.

The combined prevalence and 95%CI of MS in Brazilian adolescents, obtained from studies that used the IDF criteria, are shown in Figure 2. For this analysis, data from Kuschnir et al.’s^23^ study were extracted by Brazilian regions, thus configuring 12 studies.

Heterogeneity among the studies (Tau^2^=0.072; I^2^ 85.1%; p*<*0.010) was partially explained by the differences in MS prevalence among Brazilian regions (Q=24.7; p<0.001). The lowest MS prevalence (95%CI) was determined for North Region of Brazil, 1.8% (1.52–2.13), and the highest prevalence was determined for Northeast Region of Brazil, 2.9% (2.62–3.23). South, Southeast, and Midwest Regions of Brazil showed MS prevalence (95%CI) as follows: 2.7% (1.68–4.37), 2.6% (2.26–2.89), and 2.9% (2.48–3.35), respectively.

An influence analysis identified the study of Kuschnir et al.^23^ for the South and North Regions of Brazil with a major contribution to the heterogeneity by the Baujat test,^38^ and the exclusion of these studies would lead to the determination of a combined prevalence of 2.7% (95%CI 2.56–2.94) and absence of heterogeneity.

The prevalence of MS in males was 2.9% (95%CI 2.65–3.18) and that in females was 2.4% (95%CI 1.90–2.90), with a significant difference by the chi-square test (p<0.001). It was not possible to determine the combined prevalence of MS according to nutritional status; however, as it can be seen in Table 3, the prevalence of overweight/obesity in the studies included in the meta-analysis varied between 14.5 and 27.8%, as determined in descriptive analyses in those studies.

**Table 3:** Prevalence/confidence interval of metabolic syndrome components in Brazilian adolescents.

Study	Prevalence (%) or 95%CI* of MS components					Nutritional status (%)
	AO	High BP	HG	Low HDL-C	HTG	
Quintão et al.^17^	1.4 (males) 4.0 (females)	16.7 (males) 3.0 (females)	0 (males) 1.0 (females)	30.6 (males) 35.0 (females)	0 (males) 4.0 (females)	OW: 11.6 OB: 2.9
Alvarez et al.^18^	9.0 (6.30–12.90)	12.5 (7.90–17.05)	22.3 (13.30–31.70)	32.5 (23.60–41.40)	3.7 (1.90–5.40)	OW–OB: 15.8
Stabelini Neto et al.^19^	Not informed	18.9	4.7	29.2	18.3	LW: 5.2 ET: 77.8 OW: 11.0 OB: 6.0
de Sousa et al.^20^	46.8	18.4	16.0	54.0	27.6	Not informed
Furtado Neto e Ribeiro^21^	12.2	12.2	0.40	37.4	17.7	LW: 30.3 ET: 33.1 OW: 11.1 OB: 25.4
Granjeiro et al.^22^	2.0 (0.64–4.70)	12.9 (8.76–18.03)	4.5 (2.19–8.07)	23.3 (17.83–29.47);	6.9 (3.99–11.09)	OW–OB: 14.4
Kuschnir et al.^23^	12.6 (11.60–13.70)	8.2 (7.60–8.90)	4.1 (3.50–4.80)	32.7 (30.30–35.20)	4.6 (4.10–5.10)	Not informed
Assis et al.^24^	19.9 (15.52–24.82)	7.6 (4.89–11.21)	2.9 (1.37–5.58)	23.5 (18.84–28.70)	8.0 (5.16–11.59)	OB: 27.5
Pani et al.^25^	11.3	1.9	7.5	22.6	20.7	OW: 17.0 OB: 7.5
dos Santos et al.^26^	15.3	8.8	5.1	25.2	6.6	LW: 6.6 ET: 67.5 OW:15.3 OB: 10.6
Reuter et al.^28^	7.6	18.7	14.1	3.3	4.7	LW–ET: 72.2 OW: 16.6 OB: 11.2
Guilherme et al.^29^	Not informed	15.4	12.4	Not informed	Not informed	Not informed
Lustosa et al.^30^	11.9	5.2	18.6	50.5	4.3	LW: 2.5 ET: 80.7 OW: 12.5 OB: 4.3

* Some studies showed the prevalence of components MS, whereas others showed the prevalence and confidence interval for those components. MS: metabolic syndrome; 95%CI: confidence interval; AO: abdominal obesity; BP: blood pressure; HG: hyperglycemia; HDL-C: high-density lipoprotein cholesterol; HTG: high serum triglyceride; OW: overweight; OB: obesity; LW: low weight; ET: eutrophy.

The presence of publication bias was analyzed, and the asymmetry of the funnel plot was not shown to be significant (p=0.450).

The prevalence of MS components was determined from eight studies as follows (prevalence, 95%CI, I^2^, p): abdominal obesity (0.11, 0.08–0.15, 94.5%, <0.001), low HDL (0.22, 0.13–0.36, 94.5%, <0.001); high TG (0.22, 0.13–0.36, 94.5%, <0.001), arterial hypertension (0.10, 0.08–0.14, 93.7%, <0.001), and high blood glucose (0.09, 0.05–0.14, 98.3%, <0.001).

The results suggest a higher prevalence for low HDL, followed by abdominal obesity and arterial hypertension. However, these results must be taken with care due to the high heterogeneity found.

Heterogeneity among studies used to determine the prevalence of abdominal obesity can be partially explained, as demonstrated by a subgroup analysis, by the measurement method of waist circumference (p=0.022). There was no significant difference in determining the prevalence of arterial hypertension, in a subgroup analysis, between studies that used different blood pressure measurement devices (p=0.495).

## DISCUSSION

This systematic review and meta-analysis included more than 40,000 adolescents enrolled in public and private schools in all Brazilian regions, and they allowed the determination of MS prevalence and its components from 2010 to July 2021.

The combined MS prevalence determined in this study was 2.7, 4.5, and 12.5%, considering the IDF,^6^ Cook et al.’s,^35^ and de Ferranti et al.’s^36^ criteria, respectively. Accordingly, Tavares et al.^11^ reported MS prevalence in Brazilian adolescents varying, according to MS definition criteria, between zero and 11.9%, from 1990 to 2010, considering population-based studies. Bitew et al.^39^ reported prevalence in children and adolescents in developing countries of 4.0% (IDF criteria) and 8.2% (Ferranti et al.).^36^ Results of meta-analysis conducted in Chinese adolescents also showed a low MS prevalence (1.8%) using IDF criteria.^40^ These differences can be accounted for the lower concordance between different criteria applied to adolescents of normal weight, in comparison with the group of overweight and obese adolescents.^41–43^

Regarding the distribution by sex, we showed a higher prevalence of MS in male adolescents using IDF criteria. Similar results were found by Bitew et al.^39^ and Ye et al.^40^ A possible justification for this result is the higher prevalence of obesity among male adolescents, one of the main risk factors for MS.^44–47^ These differences may be associated with divergent behavior patterns, such as longer time spent on watching television per week and, consequently, less time spent on physical activity, in addition to greater consumption of sugary drinks and unhealthy snacks by boys.^48,49^

The results of our study showed a higher prevalence of low HDL-C, followed by abdominal obesity and arterial hypertension. Similar results were verified in the meta-analysis by Bitew et al.,^39^ where low HDL-C was also the most prevalent component among children and adolescents. In this regard, a systematic review conducted by Silva et al.^50^ to assess adolescent eating patterns pointed out that the most frequent pattern in this population, regardless of the country studied, is the “Western pattern diet,” characterized by high consumption of whole milk products, foods with a high content of simple sugar and fat, fast foods, and soft drinks. Some studies included in that review observed a positive association between this dietary pattern and changes in the lipid profile, such as high concentrations of total cholesterol, TGs, and low-density lipoprotein (LDL)-cholesterol and reduced HDL-C.

A limitation of our systematic review and meta-analysis refers to the high heterogeneity among the studies that may have influenced the results. In this regard, subgroup analysis according to Brazilian regions was carried out, showing a significant difference; and influence analysis demonstrated that the southern and northern regions had a greater contribution to the verified heterogeneity. Kuschnir et al.^23^ attributed the higher prevalence of MS found in the South region in their study to different eating habits and lifestyles in relation to other regions, since these are the main factors in the genesis of obesity, which is a central component in MS diagnosis using IDF criteria. Considering North Region of Brazil, the authors also demonstrated great variation in MS prevalence, presenting the Brazilian capitals with the highest and lowest prevalence.

Another potential source of heterogeneity found in this meta-analysis was the variation in the proportions of individuals classified as eutrophic or overweight/obese across individual studies, since a higher prevalence of MS is shown for the last group.^51,52^ However, it was not possible to conduct this analysis due to the small number of studies that reported the prevalence of MS in these different subgroups. It is also noteworthy that few studies described the detailed sociodemographic characteristics of the adolescents and specified the prevalence of MS in adolescents by type of school (public or private), which made a more detailed analysis of these aspects unfeasible.

Regarding the study quality assessment, possible limitations in individual studies, including methodological variations and low clarity in the description of the methods of collection and/or analysis of anthropometric data, were shown not to significantly influence the results of the present study in sensitivity analyses. These analyses increase the reliability of our results. This is the first systematic review and meta-analysis on the prevalence of MS in Brazilian adolescents.

This study allowed the determination of MS prevalence in Brazilian adolescents, showing a higher prevalence in males, and the determination of the most prevalent MS components in this population, identifying relevant aspects to be addressed for the prevention of associated comorbidities that have important impact on public health.
